# Pre-Ischemic Treadmill Training for Prevention of Ischemic Brain Injury via Regulation of Glutamate and Its Transporter GLT-1

**DOI:** 10.3390/ijms13089447

**Published:** 2012-07-26

**Authors:** Xiaojiao Yang, Zhijie He, Qi Zhang, Yi Wu, Yongshan Hu, Xiaolou Wang, Mingfen Li, Zhiyuan Wu, Zhenzhen Guo, Jingchun Guo, Jie Jia

**Affiliations:** 1 Department of Rehabilitation, Huashan Hospital, Fudan University, Shanghai 200040, China; E-Mails: xj8842436@163.com (X.Y.); he_zhijie@hotmail.com (Z.H.); friday0451@163.com (Q.Z.); wuyi4000@163.com (Y.W.); drhuys@sina.com.cn (Y.H.); daidaixiaomi@yeah.net (M.L.); wuzhiyuan428@163.com (Z.W.); guozhenzhen0908@yahoo.com.cn (Z.G.); 2 State Key Laboratory of Medical Neurobiology, Fudan University, Shanghai 200032, China; 3 Department of Sports Medicine and Rehabilitation, Medical College of Fudan University, Shanghai 200032, China; 4 The Third Teaching Hospital of Xinxiang Medical University, Xinxiang 453003, China; E-Mail: star19870220@163.com

**Keywords:** glutamate, glutamate transporter-1 (GLT-1), pre-ischemic treadmill training, ischemia, neuroprotection

## Abstract

Pre-ischemic treadmill training exerts cerebral protection in the prevention of cerebral ischemia by alleviating neurotoxicity induced by excessive glutamate release following ischemic stroke. However, the underlying mechanism of this process remains unclear. Cerebral ischemia-reperfusion injury was observed in a rat model after 2 weeks of pre-ischemic treadmill training. Cerebrospinal fluid was collected using the microdialysis sampling method, and the concentration of glutamate was determined every 40 min from the beginning of ischemia to 4 h after reperfusion with high-performance liquid chromatography (HPLC)-fluorescence detection. At 3, 12, 24, and 48 h after ischemia, the expression of the glutamate transporter-1 (GLT-1) protein in brain tissues was determined by Western blot respectively. The effect of pre-ischemic treadmill training on glutamate concentration and GLT-1 expression after cerebral ischemia in rats along with changes in neurobehavioral score and cerebral infarct volume after 24 h ischemia yields critical information necessary to understand the protection mechanism exhibited by pre-ischemic treadmill training. The results demonstrated that pre-ischemic treadmill training up-regulates GLT-1 expression, decreases extracellular glutamate concentration, reduces cerebral infarct volume, and improves neurobehavioral score. Pre-ischemic treadmill training is likely to induce neuroprotection after cerebral ischemia by regulating GLT-1 expression, which results in re-uptake of excessive glutamate.

## 1. Introduction

Advances in clinical and emergency medicine have drastically decreased the morbidity and mortality of stroke patients that receive immediate medical attention. Despite dramatic improvements in treatment, strokes often lead to a significant decrease in patients’ life quality, which results from lasting impairments in cognitive, motor, and sensory functions. Due to the potential impairments and the limitation of treatments, effective prevention remains an optimal approach to improve prognosis in stroke [1]. Exercise has been shown to reduce ischemic injury, and be beneficial in learning and memory [2]. Treadmill training which was used as a preventive intervention in rats has been previously demonstrated to alleviate neuronal damage after cerebral ischemia [3], maintain neurovascular integrity [4], improve blood-brain barrier function, and alleviate brain edema [5]. In addition, the pre-conditioning provided by regular exercise can reduce brain inflammation after cerebral ischemic injury [6].

The excessive release of glutamate, the primary excitatory neurotransmitter throughout the central nervous system (CNS) after cerebral ischemia-reperfusion injury can induce neurotoxicity. This increased toxicity is responsible for the majority of stroke-associated brain injuries [7]. It has been demonstrated that the CNS accurately regulates extracellular glutamate, effectively prevents abnormal neural signal transduction and excitatory toxicity [8]. The abundance of extracellular glutamate is dual-regulated by the direct release and re-uptake. Unfortunately, no related extracellular enzymes exist to decrease excessive glutamate concentration, while the maintenance of extracellular glutamate levels solely is reliant on the function of excitatory amino acid transporters (EAATs). EAATs are specialized membrane-bound secondary transport molecules that bear a resemblance to common ion channels. A total of 5 types of Na-dependent EAATs with a high affinity for glutamate have been identified in the mammalian CNS [9], including glutamate-aspartate transporter (GLAST), glutamate transporter-1 (GLT-1), excitatory amino acid carrier (EAAC-1), EAAT-4, and EAAT-5. Of these, GLT-1 primarily expresses in glial cell membranes [10], and thus plays the most important role in elimination of extracellular glutamate in nerve cells [11].

Pre-ischemic treadmill training up-regulates the concentration of gamma-aminobutyric acid (GABA) and down-regulates the expression of glutamate receptors NR2B and mGluR5 [12,13], resulting in reduced neurotoxicity and induced cerebral protection after cerebral ischemia. The true role of EAATs in the improved cerebral ischemic tolerance induced by pre-ischemic treadmill training remains unclear, though a myriad of evidence in recent decades suggests that EAATs are crucial to this mechanism.

The GLT-1 transporter is crucial to glutamate regulation. It has been demonstrated that ischemic/hypoxic pre-conditioning induces cerebral ischemic tolerance by up-regulating the GLT-1 expression [14,15]. Thus, reduction of excess glutamate concentration after ischemic stroke maybe due to the change of GLT-1 level regulated by pre-ischemic treadmill training. In order to assess the validity of this hypothesis, the present study used a rat model of middle cerebral artery occlusion (MCAO) with two weeks of pre-ischemic treadmill training compared with non-trained rats in order to determine the extracellular glutamate concentration and the expression of GLT-1 protein in the brain. Furthermore, neurobehavioral score and cerebral infarct volume were detected in order to test brain damage.

## 2. Results and Discussion

### 2.1. Extracellular Glutamate Concentration

**Figure 1:**
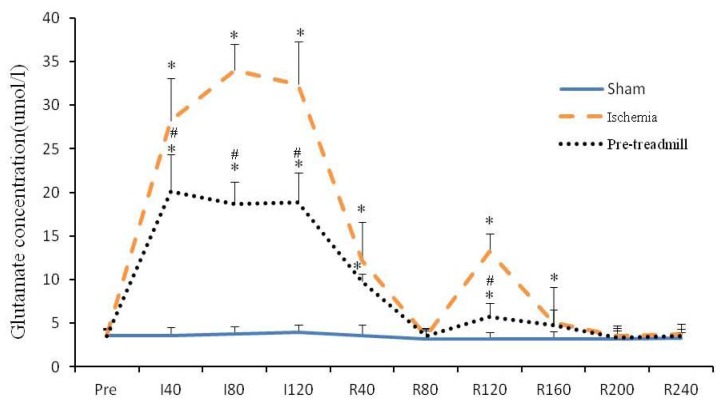
Changes in glutamate concentration in the striatal dialysate during ischemia/reperfusion. Ranges shown on the x-axis correspond to conditions as follows: before ischemia (Pre); during ischemia (I40–I120); and after reperfusion (R40–R120). The glutamate concentrations in the pre-treadmill group at various time intervals were significantly lower than those in the ischemic group. * *p* < 0.05 *vs*. the sham group; ^#^ *p* < 0.05 *vs*. the ischemic group.

A similar trend in extracellular glutamate concentration variation was observed in the ischemic group and the pre-treadmill group, as shown in Figure 1. Furthermore, the extracellular glutamate concentration increased in both groups after 40 min of ischemia and remained at elevated levels until 120 min. The glutamate concentration initially began to decrease after reperfusion, reaching the normal level 80 min after reperfusion. A second increase was observed at 120 min, peaking at 160 min and decreasing to normal levels after 200 min. The level continued to remain normal until 240 min. Extracellular glutamate concentrations were notably lower in the pre-treadmill group at all observed times compared with the other groups, and peak concentrations in the pre-treadmill group were dramatically lower than those observed in the ischemic group.

### 2.2. GLT-1 Protein Expression

**Figure 2:**
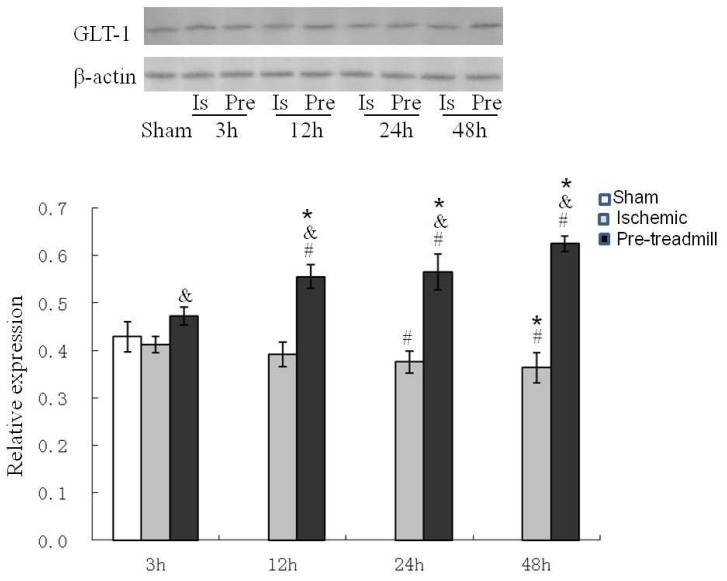
Western blot analysis of GLT-1 expression. The expression of the GLT-1 protein in the pre-treadmill group was significantly higher than that in the ischemic group. Grey-value analysis was completed using β-actin as the internal reference, and the results were expressed as mean ± SD. The experimental groups are represented by abbreviations as follows: sham group (Sham), ischemic group (Is), and pre-treadmill group (Pre). *p* < 0.01 *vs*. the ischemic group at various time points; * *p* < 0.05 *vs*. the same group at 3 h; # *p* < 0.01 *vs*. the sham group.

Repeated measures analysis of variance (ANOVA) was performed on the ischemic group and pre-treadmill group to calculate the main effect by group (*F* = 715.596, *p* < 0.01), main effect by time (*F* = 7.570, *p* = 0.002), and the interaction between group and time (*F* = 29.028, *p* < 0.01). After group stratification, repeated measures ANOVA was completed, and the GLT-1 expression at different time intervals was compared using the Bonferroni test. The GLT-1 expression exhibited a declining trend with time in the ischemic group, as shown in Figure 2; however, a significant difference in GLT-1 expression was detected 48 h after ischemia compared with that observed at 3 h (*p* < 0.05). GLT-1 expression exhibited an increasing trend with time in the pre-treadmill group, and expression levels were significantly higher at 12, 24, and 48 h after treadmill training compared with that observed at 3 h (*p* < 0.05). GLT-1 expression reached its highest level at 48 h. Following time stratification, GLT-1 expression levels were observed to be significantly lower at 3, 12, 24, and 48 h in the pre-treadmill group compared with those observed in the ischemic group (*p* < 0.01), as revealed by ANOVA or *t*-test.

### 2.3. Neurobehavioral Score

**Figure 3:**
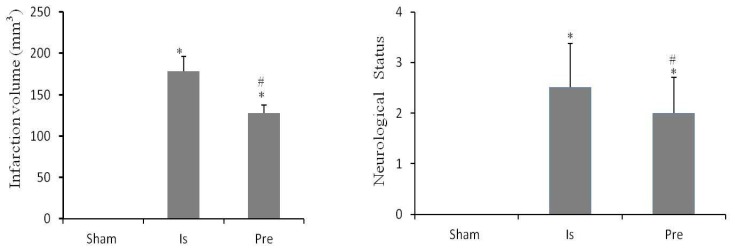
Neurobehavioral scores and cerebral infarct volume after middle cerebral artery occlusion. The neurobehavioral scores and cerebral infarct volume were all 0 in the sham-operation group. In comparison with those in the ischemic group, the rats in the pre-treadmill group exhibited a significant improvement in neurobehavioral score and cerebral infarct volume. The experimental groups are represented by abbreviations as follows: sham group (Sham), ischemic group (Is), and pre-treadmill group (Pre). * *p* < 0.01 *vs*. the sham-operation group; # *p* < 0.05 *vs*. the ischemic group. All data are described as mean ± SD, *n* = 6.

The neurobehavioral score was assessed after 24 h of ischemia, as shown in Figure 3. The neurobehavioral score was 2.33 ± 0.82 in the ischemic group and 2.20 ± 0.63 in the pre-treadmill group, indicating significant improvement following pre-ischemic treadmill training.

### 2.4. Cerebral Infarct Volume

Rats were sacrificed after neurobehavioral scoring, and variations in infarct volume were investigated, as shown in Figure 3. The cerebral infarct volume in the pre-treadmill group was 121.4 ± 7.5 mm^3^, significantly lower than that observed in the ischemic group (177.8 ± 14.3 mm^3^). No significant ischemic injury (0.0 ± 0.0 mm^3^) was observed in the sham-operation group.

### 2.5. Discussion

The amino acid glutamate is an excitatory neurotransmitter present in the central nervous system (CNS). After cerebral ischemia-reperfusion injury, the neurotoxicity induced by excessive release of glutamate is one of the major causes. The uptake of excess extracellular glutamate by glutamate transporters normally maintains low glutamate concentration in the extracellular fluid. Inhibition of glutamate uptake with application of an EAAT inhibitor produces significantly greater increase in glutamate concentration to as much as 65% [8]. In the EAATs family, GLAST and GLT-1 are central to the maintenance of normal CNS function. GLT-1 expresses in astrocytes of various brain regions particularly in cerebral cortex and hippocampus [16] and accounts for 90% of glutamate re-uptake in the prosencephalon of adult rats [17]. GLT-1-knockout mice reveal selective neuronal deterioration in the hippocampal CA1 region, which demonstrates the roles of GLT-1 in neuroprotection [18].

Up-regulation of glial GLT-1 expression has been demonstrated to induce cerebral ischemic tolerance in rats [15,19]. By contrast, Kosugi and Kawahara demonstrated that down-regulation and reverse transport of GLT-1 induced by preconditioning in neuron/astrocyte co-cultures led to minor increase in extracellular glutamate concentration, which induced ischemic tolerance via the NMDA receptor pathway [20]. These studies, however, were conducted in vitro and the effect of preconditioning on GLT-1 expression in vivo remains unclear. The present in vivo study showed that compared with ischemic group, glutamate concentration was significantly reduced during early stages in pre-treadmill group. We also detected that levels of glutamate concentration achieved the lowest at 80 min after reperfusion and formed the second peak probably associating with the reperfusion injury from 80 min to 240 min after reperfusion. On the basis of previous research exhibiting that GLT-1 expression reduces after ischemic-hypoxic insults [21], we observed that GLT-1 expression at 3, 12, 24, and 48 h significantly increased in the pre-treadmill group. Our results are consistent with previous studies demonstrating that GLT-1 expression was significantly up-regulated within the initial 6 h after ischemic preconditioning [22]. It may be due to post-ischemic gliosis in early ischemia after pre-treadmill training. With the ischemic time passing by, GLT-1 expression in the ischemic group showed a gradual decreasing trend, while GLT-1 expression exhibited a marked increasing trend in the pre-treadmill group. Improvements in both neurological function and cerebral infarct volume indicated that alterations in GLT-1 expression and glutamate concentration might be correlated with the neuroprotection. Thus, pre-ischemic treadmill training may down-regulate glutamate concentration thereby reducing excitatory toxicity through the up-regulation of GLT-1 expression to induce ischemic tolerance. Ischemic preconditioning has been shown to play a role in neuroprotection because of the up-regulation of adhesive proteins expression in GLT-1 of the hippocampus CA1 region enhancing the maximum affinity of GLT-1 within 5 days after ischemia and thus increasing glutamate re-uptake by GLT-1. This contributes to alleviating delayed neuron death [14]. It is similar to the observation in the present study that pre-ischemic treadmill training up-regulates the GLT-1 expression at 12, 24, and 48 h after ischemia and plays an important role in neuroprotection.

Spliced variants for GLT-1 have been reported in some research [16,23,24], the expression of which is mutated in the brains of mice subjected to chemical hypoxia. Among these spliced variants, mGLT-1/5UT4 and mGLT-1/5UT5 transcripts were increased transiently in the frontal cortex, whereas a down-regulated expression of these transcripts was found in the hippocampus 12–72 h after hypoxic neuronal damage in a model. Meanwhile, a significantly up-regulated expression of mGLT-1/5UT4 and a down-regulation of mGLT-1/5UT5 were detected in the cerebellum before returning to normal, whereas the expression of mGLT-1/5UT3 showed to be regulated in the cerebellum only [23]. In addition, hypoxia was proved to induce the expression of a splice variant of GLT-1 in neurons of pigs [24]. However, further studies should be carried out to investigate the effect of pre-ischemic treadmill training on GLT-1 functions and its spliced variants in addition to up-regulation of GLT-1 expression.

The excessive glutamate was converted to glutamine via the re-uptake by glutamate transporter and glutamate-glutamine cycle, which reduced neurotoxicity after ischemia. An effective glutamate-glutamine cycle in glial cells depends on the regulated coordination between glutamate uptake and glutamate degradation [25]. As mentioned above, GLT-1 plays an important role in re-uptake of excessive glutamate. Thomas Rauen *et al.* proved that the transcriptional regulation of the key proteins such as GLAST and glutamine synthetase in the glial portion of the glutamate-glutamine cycle may have an effect on transmitter clearance and transmitter recycling [25]. In this study, the results showed that pre-ischemic treadmill training could up-regulate GLT-1 expression after cerebral ischemia.

Moreover, the re-uptake of glutamate by the glutamate transporter is dependent on the Na+ electrochemical gradient, which is an energy consuming process. The co-compartmentalization of GLT-1, Na+-K+-ATP, glycolytic enzyme, and the mitochondrion provides the major mechanism of energy supply for transporting glutamate [26]. Previous studies have demonstrated that exercise induces mitochondrial biogenesis after cerebral ischemia injury [27], improves capillary generation and cortical blood flow volume in rats [28] and increases the number of cerebral small vessels in aged, healthy subjects [29]. Thus, improvement of cerebral blood flow during stages of ischemia may be a better way to promote functional recovery. Moreover, it could associate with the regulation Na^+^-K^+^-ATP, mitochondrial biogenesis, enhancing transportation of glutamate via the glutamate transporter and down-regulation of excessive glutamate.

The present study provides a valuable initial assessment of pre-treadmill training for prevention of ischemic injury. However, it is primarily limited because the determination of GLT-1 expression only considers the time-dependent changes after reperfusion; the corresponding GLT-1 expression during ischemic stage was not detected. In addition, there are limitations in the TTC method which cause uncertainties in the measurement of cerebral infarct volume due to marked metabolic changes over the course of evolution of brain infarction [30]. We will choose more accurate detection methods to reflect real brain injury levels in the future.

## 3. Experimental Section

### 3.1. Animals and Groups

Male Sprague-Dawley (SD) rats, each weighing 250–280 g, were purchased from the Shanghai Laboratory Animal Center (SLAC), Chinese Academy of Sciences. Animals were provided with free access to food and water. Furthermore, animals were placed on a 12/12 h light/dark cycle. Rats were randomly divided into 3 equal groups: the pre-treadmill group, the ischemic group, and the sham group. All of the 3 groups rats were subjected to adaptive running at a speed of 5–8 m/min for 30 min per day for 2 days, as previously described [12,31]. Rats in the pre-treadmill group started training on an electric treadmill machine (DSPT-202 Type 5-Lane Treadmill; Litai Biotechnology Co., Ltd., Hangzhou, China) at a 0° slope and speed of 20 m/min for 30 min per day for 2 weeks, 5 days per week before ischemia. Rats in the sham and ischemic groups were not exposed to the treadmill before sham or MCAO surgery, but remained in their home cages for 2 weeks.

### 3.2. Establishment of Rat Model of Middle Cerebral Artery Occlusion (MCAO)

Rats were peritoneally injected with 10% chloral hydrate at a dose of 360 mg/kg using the modified Longa’s method [32] to establish the rat model of left MCAO. Briefly, a surgical nylon monofilament with a silicone tip of 0.36 ± 0.02 mm diameter (Beijing Shadong Biotech Co., Ltd., Beijing, China) was immersed in heparin in advance and was inserted from the external carotid artery into the lumen of the internal carotid artery, finally the nylon monofilament with a length of approximately 1.8–2.0 cm was placed into the cranium in order to cause MCAO. Regional blood flow on the left side of the brain was detected using a laser Doppler velocimeter (LDPM, PeiFlux5000, Perimed, Jarfalla, Sweden) [33]. A circulating heating pad was used during the whole surgical procedure to maintain the rectal temperature of rats at 37 °C. After 120 min of cerebral ischemia, the nylon monofilament was removed surgically and reperfusion was performed. Rats in the sham group were performed the same surgical procedure but without MCAO.

### 3.3. Microdialysis Sampling

Microdialysis sampling was completed as previously described [12]. Artificial cerebrospinal fluid containing 7.605 g/L NaCl, 0.22 g/L KCl, 0.144 g/L CaCl_2_, 0.162 g/L MgCl_2_·6H_2_O, 2.10 g/L NaHCO_3_, 0.014 g/L Na_2_HPO_4_·12H_2_O, and 0.072 g/L NaH_2_PO_4_·2H_2_O was used as the intracerebral microdialysis perfusate and then injected using an MD21001 power micro-injector. After being subjected to anesthesia, rats were placed in the prone position. The head was fixed on a stereo positioning instrument in the horizontal position, and microdialysis perfusate samples were taken from rat brain while specimens remained under anesthesia.

The balanced microdialysis probe was implanted into the brain of rat specimens through the previously indwelling tubes, and the microdialysis sensor (effective dialysis membrane length of 4 mm; MAB 6.14.4 sensor; stainless steel) was implanted into the brain through the indwelling tube. The entrance of the sensor was connected with the syringe on the microdialysis pump (MD-0100; Bioanalytical system, West Lafayette, IN, USA) via a plastic catheter, and the outlet was connected with a centrifugal tube (0.5 mL) via the catheter to collect dialysate for the determination of glutamate using high performance liquid chromatography (HPLC). Following that the system was balanced at 1 μL/min for 90 min. All outflow fluid was collected in a microtube for an additional 30 min, and all outflow fluid was subsequently collected into another tube.

Control fluid was collected by performance of microdialysis at a flow speed of 2 μL/min applied to the rostral ventrolateral medulla (rVLM), resulting in the collection of 20 μL of dialysate during each collection round. The changes in glutamate concentration in the dialysate were observed by collection of the dialysate at 40, 80, and 120 min after the suture line was placed and at 40, 80, 120, 160, 200, and 240 min after reperfusion. A total of 9 tubes of dialysate were collected during the entire experiment. After the completion of animal experiments, the changes in glutamate concentration in each dialysate sample were determined using the HPLC-fluorescence detection method.

### 3.4. Determination of Glutamate Concentration

The concentration of glutamate was determined according to the method described previously [13], and the amino acid neurotransmitter was isolated using HPLC. The chromatographic conditions were set as follows. The chromatographic column used was a Hypersil ODS23 4.6 mm × 250 mm, 5 μm (GL Co., Ltd., Tokyo, Japan), and the fluorescence detector was equipped with 3D chromatography workstation. The mobile phase used was KH_2_SO_4_ (0.1 mol/L, pH 6.0): methanol: acetonitrile (*v*/*v*) = 6:3:1. The acetonitrile was filtered using a filtration membrane with a diameter of 0.45 μm and gas-separated for 15 min prior to the experiment, and the mobile phase was prepared as used. The excitation wavelength of fluorescence detection (Ex) was 340 nm, the emission wavelength (Em) was 455 nm, the flow velocity was 1 mL/min, and the column temperature was 40 °C. The entire duration of the chromatographic procedure from start to final elution was 25 min.

The derivative agent and pre-column derivatization were prepared as follows. Approximately 13.4 mg of *O*-phthalaldehyde (OPA) was dissolved in 1 mL of ethanol and subsequently combined with 20 μL of 2-mercaptoethanol (2-MCE). This step was followed by the addition of 4 mL of sodium tetraborate buffer solution (0.1 mol/L, pH 9.6). The resultant solution was sealed tightly and stored at a low temperature. Two days later, 20 μL of 2-MCE was added. A total volume of 40 μL of the standard solution (10 mmol/L glutamate solution) was added to the samples after preconditioning with 20 μL of the OPA derivative agent. The mixture was gently mixed and allowed to settle for 2 min. A volume of 20 μL of the mixture was injected for HPLC analysis. Retention time method was used for qualitative analysis, and peak-area internal standard method was employed for quantitative analysis.

### 3.5. Western Blot Analysis

Striatal brain tissues were collected for Western blot analysis at 3, 12, 24, and 48 h (including 2 h ischemic) after ischemia/reperfusion. About 10 μg of protein was separated using 12% SDS-PAGE, transferred onto a PVDF membrane, and blocked in 5% non-fat milk at room temperature for 2 h. Resultant protein was incubated in rabbit anti-GLT-1 (Santa Cruz Biotechnology, Inc., Santa Cruz, California, USA) and β-actin (Santa Cruz Biotechnology, Inc., Santa Cruz, California, USA), followed by incubation in HRP-labeled goat anti-rabbit IgG (1:5000, Zymed) at room temperature for 2 h. The resultant protein was visualized using the enhanced chemiluminescence method (Beyotime) and analyzed with a gel imaging analysis system.

### 3.6. Neurobehavioral Score

Neurobehavioral changes were assessed using the 5-point scoring system after 120 min of ischemia followed by 24 h of reperfusion in rats [34], as shown in Figure 3. A score of 0 (Score 0) indicated no apparent symptoms in the neurological system; a score of 1 (Score 1) indicated that the contralateral forepaw failed to stretch completely; a score of 2 (Score 2) indicated the behavior of crawling in circles and dumping to the contralateral side; a score of 3 (Score 3) indicated no independent walking; and a score of 4 (Score 4) indicated a complete loss of consciousness.

### 3.7. Measurement of Cerebral Infarct Volume

After neurobehavioral scoring, rats were anesthetized with 10% chloral hydrate, and brain tissues were rapidly collected, as shown in Figure 3. Sample tissues were stored at −20 °C for 10 min, and they were then cut into 6 coronal sections from cerebral frontal pole to occipital pole, each with a thickness of 2.0 mm. Brain tissue sections were immersed in phosphate buffer solution (pH 7.4) containing 2% TTC at 37 °C for 30 min, fixed in 4% paraformaldehyde for 24 h [30], and photographed using a digital camera (DC240; Kodak, New York, NY, USA). The cerebral infarct volume was calculated using the NIH image analyzer software [34].

### 3.8. Statistical Analysis

All statistical analyses were performed using the statistical software SPSS 16.0 (SPSS Inc.: Chicago, IL, USA), and all data were expressed as mean ± standard deviation (mean ± SD). One-way ANOVA was used to compare groups, and the difference within groups was tested for statistical significance using the repeated measures ANOVA. Experimental differences across the study were initially tested using repeated measures ANOVA. Subsequently, the differences between groups after time stratification were tested for significance using ANOVA or *t* test. The differences between groups after group stratification were tested for significance using repeated measures ANOVA. The differences at two time points were compared using the Bonferroni method, and the differences within groups were compared using Tukey method. A *p* value less than 0.05 (*p* < 0.05) was considered to be statistically significant.

## 4. Conclusions

Pre-ischemic treadmill training down-regulates the level of extracellular glutamate concentration in the striatum of rats after cerebral ischemia and promotes the GLT-1 expression, accompanied by an improvement of neurological status and cerebral infarct volume. Pre-ischemic treadmill training may play a neuroprotective role in reducing neurotoxicity via promoting the expression of GLT-1 to regulate glutamate concentration. This study will help to clarify the neuroprotection mechanisms of the pre-ischemic treadmill training by regulating the glutamate system. Further investigation of this mechanism is critical to contributing to the effective clinical treatment of ischemic stroke.

## References

[1] Goldstein L.B., Bushnell C.D., Adams R.J., Appel L.J., Braun L.T., Chaturvedi S., Creager M.A., Culebras A., Eckel R.H., Hart R.G. (2011). Guidelines for the primary prevention of stroke: A guideline for healthcare professionals from the American Heart Association/American Stroke Association. Stroke.

[2] Cotman C.W., Berchtold N.C., Christie L.A. (2007). Exercise builds brain health: Key roles of growth factor cascades and inflammation. Trends Neurosci.

[3] Yang Y.R., Wang R.Y., Wang P.S., Yu S.M. (2003). Treadmill training effects on neurological outcome after middle cerebral artery occlusion in rats. Can. J. Neurol. Sci.

[4] Ding Y.H., Ding Y., Li J., Bessert D.A., Rafols J.A. (2006). Exercise pre-conditioning strengthens brain microvascular integrity in a rat stroke model. Neurol. Res.

[5] Guo M., Cox B., Mahale S., Davis W., Carranza A., Hayes K., Sprague S., Jimenez D., Ding Y. (2008). Pre-ischemic exercise reduces matrix metalloproteinase-9 expression and ameliorates blood-brain barrier dysfunction in stroke. Neuroscience.

[6] Curry A., Guo M., Patel R., Liebelt B., Sprague S., Lai Q., Zwagerman N., Cao F.X., Jimenez D., Ding Y. (2010). Exercise pre-conditioning reduces brain inflammation in stroke via tumor necrosis factor-alpha, extracellular signal-regulated kinase 1/2 and matrix metalloproteinase-9 activity. Neurol. Res.

[7] Guyot L.L., Diaz F.G., O’Regan M.H., McLeod S., Park H., Phillis J.W. (2001). Real-time measurement of glutamate release from the ischemic penumbra of the rat cerebral cortex using a focal middle cerebral artery occlusion model. Neurosci. Lett.

[8] Hinzman J.M., Thomas T.C., Quintero J.E., Gerhardt G.A., Lifshitz J. (2012). Disruptions in the regulation of extracellular glutamate by neurons and glia in the rat striatum two days after diffuse brain injury. J. Neurotrauma.

[9] Beart P.M., O’Shea R.D. (2007). Transporters for l-glutamate: An update on their molecular pharmacology and pathological involvement. Br. J. Pharmacol.

[10] Sims K.D., Robinson M.B. (1999). Expression patterns and regulation of glutamate transporters in the developing and adult nervous system. Crit. Rev. Neurobiol.

[11] Suchak S.K., Baloyianni N.V., Perkinton M.S., Williams R.J., Meldrum B.S., Rattray M. (2003). The ‘glial’ glutamate transporter, EAAT2 (Glt-1) accounts for high affinity glutamate uptake into adult rodent nerve endings. J. Neurochem.

[12] Jia J., Hu Y.S., Wu Y., Liu G., Yu H.X., Zheng Q.P., Zhu D.N., Xia C.M., Cao Z.J. (2009). Pre-ischemic treadmill training affects glutamate and gamma aminobutyric acid levels in the striatal dialysate of a rat model of cerebral ischemia. Life Sci.

[13] Zhang F., Jia J., Wu Y., Hu Y., Wang Y. (2010). The effect of treadmill training pre-exercise on glutamate receptor expression in rats after cerebral ischemia. Int. J. Mol. Sci.

[14] Liu A.J., Hu Y.Y., Li W.B., Xu J., Zhang M. (2011). Cerebral ischemic pre-conditioning enhances the binding characteristics and glutamate uptake of glial glutamate transporter-1 in hippocampal CA1 subfield of rats. J. Neurochem.

[15] Gong S.J., Chen L.Y., Zhang M., Gong J.X., Ma Y.X., Zhang J.M., Wang Y.J., Hu Y.Y., Sun X.C., Li W.B., Zhang Y. (2012). Intermittent hypobaric hypoxia preconditioning induced brain ischemic tolerance by up-regulating glial glutamate transporter-1 in rats. Neurochem. Res.

[16] Kanai Y., Hediger M.A. (2004). The glutamate/neutral amino acid transporter family SLC1: Molecular, physiological and pharmacological aspects. Pflugers Arch.

[17] Verma R., Mishra V., Sasmal D., Raghubir R. (2010). Pharmacological evaluation of glutamate transporter 1 (GLT-1) mediated neuroprotection following cerebral ischemia/reperfusion injury. Eur. J. Pharmacol.

[18] Tanaka K., Watase K., Manabe T., Yamada K., Watanabe M., Takahashi K., Iwama H., Nishikawa T., Ichihara N., Kikuchi T. (1997). Epilepsy and exacerbation of brain injury in mice lacking the glutamate transporter GLT-1. Science.

[19] Mimura K., Tomimatsu T., Minato K., Jugder O., Kinugasa-Taniguchi Y., Kanagawa T., Nozaki M., Yanagihara I., Kimura T. (2011). Ceftriaxone preconditioning confers neuroprotection in neonatal rats through glutamate transporter 1 upregulation. Reprod. Sci.

[20] Kawahara K., Kosugi T., Tanaka M., Nakajima T., Yamada T. (2005). Reversed operation of glutamate transporter GLT-1 is crucial to the development of preconditioning-induced ischemic tolerance of neurons in neuron/astrocyte co-cultures. Glia.

[21] Torp R., Lekieffre D., Levy L.M., Haug F.M., Danbolt N.C., Meldrum B.S., Ottersen O.P. (1995). Reduced postischemic expression of a glial glutamate transporter, GLT1, in the rat hippocampus. Exp. Brain Res.

[22] Zhang M., Li W.B., Geng J.X., Li Q.J., Sun X.C., Xian X.H., Qi J., Li S.Q. (2007). The upregulation of glial glutamate transporter-1 participates in the induction of brain ischemic tolerance in rats. J. Cereb Blood Flow Metab.

[23] Münch C., Zhu B.G., Leven A., Stamm S., Einkörn H., Schwalenstöcker B., Ludolph A.C., Riepe M.W., Meyer T. (2003). Differential regulation of 5′ splice variants of the glutamate transporter EAAT2 in an in vivo model of chemical hypoxia induced by 3-nitropropionic acid. J. Neurosci. Res.

[24] Pow D.V., Naidoo T., Lingwood B.E., Healy G.N., Williams S.M., Sullivan R.K.P., O’Driscoll S., Colditz P.B. (2004). Loss of glial glutamate transporters and induction of neuronal expression of GLT-1B in the hypoxic neonatal pig brain. Dev. Brain Res.

[25] Rauen T., Wiessner M. (2000). Fine tuning of glutamate uptake and degradation in glial cells: Common transcriptional regulation of GLAST1 and GS. Neurochem. Int.

[26] Genda E.N., Jackson J.G., Sheldon A.L., Locke S.F., Greco T.M., O’Donnell J.C., Spruce L.A., Xiao R., Guo W., Putt M. (2011). Co-compartmentalization of the astroglial glutamate transporter, GLT-1, with glycolytic enzymes and mitochondria. J. Neurosci.

[27] Zhang Q., Wu Y., Zhang P., Sha H., Jia J., Hu Y., Zhu J. (2012). Exercise induces mitochondrial biogenesis after brain ischemia in rats. Neuroscience.

[28] Swain R.A., Harris A.B., Wiener E.C., Dutka M.V., Morris H.D., Theien B.E., Konda S., Engberg K., Lauterbur P.C., Greenough W.T. (2003). Prolonged exercise induces angiogenesis and increases cerebral blood volume in primary motor cortex of the rat. Neuroscience.

[29] Bullitt E., Rahman F.N., Smith J.K., Kim E., Zeng D., Katz L.M., Marks B.L. (2009). The effect of exercise on the cerebral vasculature of healthy aged subjects as visualized by MR angiography. AJNR Am. J. Neuroradiol.

[30] Benedek A., Móricz K., Jurányi Z., Gigler G., Lévay G., Hársing L.G., Mátyus P., Szénási G., Albert M. (2006). Use of TTC staining for the evaluation of tissue injury in the early phases of reperfusion after focal cerebral ischemia in rats. Brain Res..

[31] Jia J., Hu Y.S., Wu Y., Yu H.X., Liu G., Zhu D.N., Xia C.M., Cao Z.J., Zhang X., Guo Q.C. (2010). Treadmill pre-training suppresses the release of glutamate resulting from cerebral ischemia in rats. Exp. Brain Res.

[32] Longa E.Z., Weinstein P.R., Carlson S., Cummins R. (1989). Reversible middle cerebral artery occlusion without craniectomy in rats. Stroke.

[33] Huang Z., Huang P.L., Panahian N., Dalkara T., Fishman M.C., Moskowitz M.A. (1994). Effects of cerebral ischemia in mice deficient in neuronal nitric oxide synthase. Science.

[34] Zhang Q., Wu Y., Sha H., Zhang P., Jia J., Hu Y., Zhu J. (2012). Early exercise affects mitochondrial transcription factors expression after cerebral ischemia in rats. Int. J. Mol. Sci.

